# Poly[(μ-benzene-1,2,4,5-tetra­carboxyl­ato)tetra­silver(I)]

**DOI:** 10.1107/S1600536809015074

**Published:** 2009-04-30

**Authors:** M. Nawaz Tahir, Orhan Atakol, Muhammad Ilyas Tariq

**Affiliations:** a Department of Physics, University of Sargodha, Sargodha, Pakistan; b Department of Chemistry, Faculty of Science, University of Ankara, Ankara, Turkey; c Department of Chemistry, University of Sargodha, Sargodha, Pakistan

## Abstract

In the centrosymmetric title compound, [Ag_4_(C_10_H_2_O_8_)]_*n*_, the benzene ring has irregular bond lengths but remains planar (r.m.s. deviation 0.0002 Å). The Ag—O bond lengths are in the range 2.153 (3)–2.615 (4) Å. The carboxyl­ate groups are oriented at dihedral angles of 26.4 (5) and 74.9 (4)° to the benzene ring. The coordination behaviour of each carboxyl­ate O atom is different: in one carboxylate, the O atoms are coordinated to a single and two Ag atoms; in the other carboxylate, the O atoms are coordinated to two and three Ag atoms. Non-classical inter­molecular C—H⋯O hydrogen bonding is present in the crystal structure. The title compound forms a three-dimensional polymeric network due to the coordination of the Ag atoms.

## Related literature

For related structures, see: Jaber *et al.* (1997 ▶); Tahir *et al.* (1996 ▶); Ülkü *et al.* (1996 ▶).
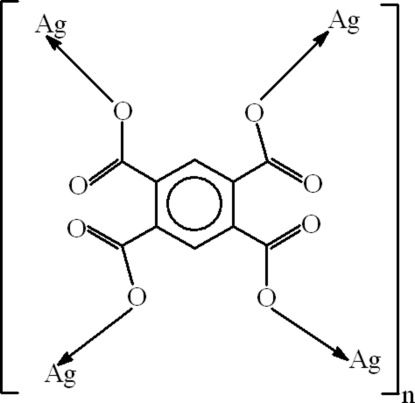


## Experimental

### 

#### Crystal data

[Ag_4_(C_10_H_2_O_8_)]
*M* *_r_* = 340.80
Monoclinic, 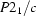

*a* = 8.328 (1) Å
*b* = 6.317 (1) Å
*c* = 10.945 (2) Å
β = 94.36 (2)°
*V* = 574.13 (16) Å^3^
*Z* = 4
Mo *K*α radiation
μ = 6.76 mm^−1^
*T* = 296 K
0.30 × 0.10 × 0.08 mm

#### Data collection

Enraf–Nonius CAD-4 diffractometer
Absorption correction: ψ scan (**MolEN**; Fair, 1990 ▶) *T*_min_ = 0.448, *T*_max_ = 0.578
1680 measured reflections
1308 independent reflections
1268 reflections with *I* > 2σ(*I*)
*R*_int_ = 0.012
3 standard reflections frequency: 120 min intensity decay: 0.1%

#### Refinement

*R*[*F* ^2^ > 2σ(*F* ^2^)] = 0.026
*wR*(*F* ^2^) = 0.089
*S* = 1.01
1308 reflections
101 parameters
H-atom parameters constrained
Δρ_max_ = 1.20 e Å^−3^
Δρ_min_ = −0.79 e Å^−3^

### 

Data collection: *CAD-4 EXPRESS* (Enraf–Nonius, 1993 ▶); cell refinement: *CAD-4 EXPRESS*; data reduction: *MolEN* (Fair, 1990 ▶); program(s) used to solve structure: *SHELXS97* (Sheldrick, 2008 ▶); program(s) used to refine structure: *SHELXS97* (Sheldrick, 2008 ▶); molecular graphics: *ORTEP-3* (Farrugia, 1997 ▶); software used to prepare material for publication: *WinGX* (Farrugia, 1999 ▶).

## Supplementary Material

Crystal structure: contains datablocks global, I. DOI: 10.1107/S1600536809015074/rk2131sup1.cif

Structure factors: contains datablocks I. DOI: 10.1107/S1600536809015074/rk2131Isup2.hkl

Additional supplementary materials:  crystallographic information; 3D view; checkCIF report

## Figures and Tables

**Table 1:** Hydrogen-bond geometry (Å, °)

*D*—H⋯*A*	*D*—H	H⋯*A*	*D*⋯*A*	*D*—H⋯*A*
C2—H2⋯O3^i^	0.93	2.5400	3.422 (6)	158

Symmetry code: (i) 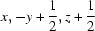.

**Fig. 1.**
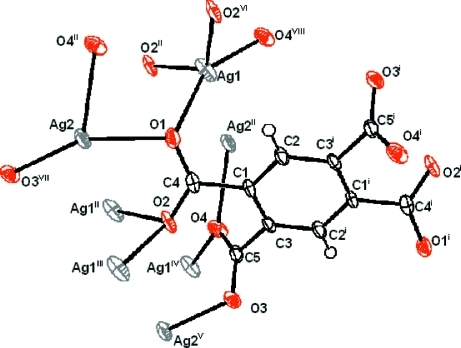
A view of part of polimeric structure of title compound with the atom numbering scheme. Displacement ellipsoids are drawn at 50% probability level. H atom is presented as a small sphere of arbitrary radius. The symmetry codes in labeling are i = -x+1, -y+1, -z+1; ii = -x, -y+1, -z+1; iii = x, -y+1/2, z-1/2; iv = x, -y+3/2, z-1/2; v = -x, y+1/2, -z+1/2; vi = x, -y+1/2, z+1/2; vii = -x, y-1/2, -z+1/2; viii = x, -y+3/2, z+1/2.

## supplementary crystallographic information

### Comment

The crystal structures of poly[bis(*p*–nitrosalicylato–O:*O*')disilver(I)] (Tahir *et al.*, 1996) and poly[bis(3,5–dinitrobenzoato–O1:O2) disilver(I)–O2:Ag;Ag':O2'] (Ülkü *et al.*, 1996) have been reported. In continuation to the interest relating to the chemistry of silver coordination with carboxylates, the title compound (I), (Fig. 1) was synthesized.

Crystal structure of silver(I) with 1,2,4,5–benzenetetracarboxylic acid (II) (Jaber *et al.*, 1997) has also been reported. The present complex (I) has been prepared by different method with same ligand as in (II). Due to the change in reaction mechanism, the coordination of O atoms of all carboxylates with Ag atoms has been affected very much. In this centrosymmetric complex, the C–O bond lengths of the carboxylato–groups vary in the range of 1.224 (3) to 1.298 (5) Å. In the benzene ring, the bond lengths of opposite sides are equal having values of 1.359 (6), 1.368 (7) and 1.414 (7) Å. The largest bond is in between the C atoms bearing carboxylate–groups. Although the bond lengths in benzene ring are irregular but it remains planar. In (I), the metallic bond is of 3.0140 (8) Å. The carboxylato–group (O1/C4/O2) makes a dihedral angle of 26.37 (51)° with the benzene ring, while the group (O3/C5/O4) is oriented at 74.90 (37)°. Non–classical intermolecular C2—H2···O3 hydrogen bond was found in the molecular structure. The crystal structure of title compound is stabilized through three dimensional polymeric network.

### Experimental

To the aqueous solution of sodium 1,2,4,5–benzenetetracarboxylate, freshly prepared solution of AgNO_3_ was added dropwise with constant stirring until the color was changed. The product was filtered and the filtrate was kept in darkness for the crystallization by slow evaporation. Needle like crystals were obtained after 72 h.

### Refinement

The H–atom was found in difference map but positioned geometrically due to the presence of heavy atoms, C—H = 0.93 Å with U_iso_(H) = 1.2U_eq_(C) and constrained to ride on the parent atom.

The maximum electron density peak appears at the fractional coordinates 0.1821 0.4015 0.7121 and is at a distance of 0.75 Å from Ag1.

### Crystal data

**Table:**

[Ag_4_(C_10_H_2_O_8_)]	*F*(000) = 628
*M**_r_* = 340.80	*D*_x_ = 3.943 Mg m^−^^3^
Monoclinic, *P*2_1_/*c*	Mo *K*α radiation, λ = 0.71073 Å
Hall symbol: -P 2ybc	Cell parameters from 25 reflections
*a* = 8.328 (1) Å	θ = 11.7–21.0°
*b* = 6.317 (1) Å	µ = 6.76 mm^−^^1^
*c* = 10.945 (2) Å	*T* = 296 K
β = 94.36 (2)°	Needle, pale yellow
*V* = 574.13 (16) Å^3^	0.30 × 0.10 × 0.08 mm
*Z* = 4	

### Data collection

**Table:**

Enraf–Nonius CAD-4 diffractometer	*R*_int_ = 0.012
ω/2θ scans	θ_max_ = 28.2°, θ_min_ = 3.7°
Absorption correction: ψ scan (*MolEN*; Fair, 1990)	*h* = 0→10
*T*_min_ = 0.448, *T*_max_ = 0.578	*k* = 0→8
1680 measured reflections	*l* = −14→14
1308 independent reflections	3 standard reflections every 120 min
1268 reflections with *I* > 2σ(*I*)	intensity decay: 0.1%

### Refinement

**Table:**

Refinement on *F*^2^	Primary atom site location: Patterson
Least-squares matrix: Full	Secondary atom site location: Difmap
*R*[*F*^2^ > 2σ(*F*^2^)] = 0.026	H-atom parameters constrained
*wR*(*F*^2^) = 0.089	*w* = 1/[σ^2^(*F*_o_^2^) + (0.0599*P*)^2^ + 3.6455*P*] where *P* = (*F*_o_^2^ + 2*F*_c_^2^)/3
*S* = 1.01	(Δ/σ)_max_ < 0.001
1308 reflections	Δρ_max_ = 1.20 e Å^−^^3^
101 parameters	Δρ_min_ = −0.79 e Å^−^^3^
0 restraints	

### Special details

**Table:**

Experimental. The structure was solved by Patterson method using *SHELX86*. The whole molecule was recognized.
Geometry. All s.u.'s (except the s.u. in the dihedral angle between two l.s. planes) are estimated using the full covariance matrix. The cell s.u.'s are taken into account individually in the estimation of s.u.'s in distances, angles and torsion angles; correlations between s.u.'s in cell parameters are only used when they are defined by crystal symmetry. An approximate (isotropic) treatment of cell s.u.s is used for estimating esds involving l.s. planes.

### Fractional atomic coordinates and isotropic or equivalent isotropic displacement parameters (Å^2^)

**Table:**

	*x*	*y*	*z*	*U*_iso_*/*U*_eq_	
Ag1	0.09967 (6)	0.44769 (6)	0.70351 (4)	0.0316 (2)	
Ag2	−0.15558 (4)	0.16471 (6)	0.44813 (3)	0.0250 (2)	
O1	0.1054 (4)	0.2412 (6)	0.5218 (3)	0.0238 (10)	
O2	0.1422 (4)	0.3437 (6)	0.3300 (3)	0.0264 (10)	
O3	0.3520 (4)	0.6473 (6)	0.1913 (3)	0.0246 (10)	
O4	0.2066 (5)	0.8108 (6)	0.3346 (3)	0.0273 (11)	
C1	0.3498 (5)	0.4182 (8)	0.4710 (4)	0.0169 (11)	
C2	0.4363 (6)	0.3358 (7)	0.5701 (4)	0.0184 (12)	
C3	0.4135 (5)	0.5821 (8)	0.4012 (4)	0.0167 (11)	
C4	0.1874 (5)	0.3273 (7)	0.4386 (4)	0.0191 (12)	
C5	0.3167 (5)	0.6850 (8)	0.2996 (4)	0.0186 (12)	
H2	0.39517	0.22806	0.61643	0.0221*	

### Atomic displacement parameters (Å^2^)

**Table:**

	*U*^11^	*U*^22^	*U*^33^	*U*^12^	*U*^13^	*U*^23^
Ag1	0.0447 (3)	0.0225 (3)	0.0247 (3)	−0.0009 (2)	−0.0155 (2)	−0.0012 (1)
Ag2	0.0254 (3)	0.0282 (3)	0.0195 (3)	−0.0047 (1)	−0.0097 (2)	0.0011 (1)
O1	0.0201 (16)	0.0262 (18)	0.0240 (17)	−0.0079 (13)	−0.0045 (13)	−0.0005 (14)
O2	0.0254 (18)	0.0292 (19)	0.0221 (17)	−0.0027 (14)	−0.0152 (14)	−0.0006 (14)
O3	0.0222 (17)	0.035 (2)	0.0156 (16)	0.0058 (14)	−0.0048 (13)	0.0024 (14)
O4	0.0291 (19)	0.033 (2)	0.0187 (16)	0.0134 (16)	−0.0057 (14)	0.0017 (14)
C1	0.0131 (18)	0.020 (2)	0.0164 (19)	−0.0001 (16)	−0.0070 (15)	0.0001 (16)
C2	0.017 (2)	0.020 (2)	0.017 (2)	−0.0003 (16)	−0.0064 (16)	0.0033 (16)
C3	0.0150 (19)	0.021 (2)	0.0130 (18)	0.0022 (16)	−0.0058 (14)	0.0001 (16)
C4	0.016 (2)	0.016 (2)	0.024 (2)	0.0017 (16)	−0.0059 (17)	−0.0050 (16)
C5	0.015 (2)	0.022 (2)	0.018 (2)	−0.0005 (16)	−0.0046 (16)	0.0013 (16)

### Geometric parameters (Å, °)

**Table:**

Ag1—O1	2.382 (3)	O2—C4	1.224 (5)
Ag1—Ag2^i^	3.0140 (8)	O3—C5	1.265 (5)
Ag1—O2^i^	2.412 (4)	O4—C5	1.293 (6)
Ag1—O2^ii^	2.314 (4)	C1—C2	1.359 (6)
Ag1—O4^iii^	2.233 (4)	C1—C3	1.414 (7)
Ag2—O1	2.311 (3)	C1—C4	1.487 (6)
Ag2—O3^iv^	2.153 (3)	C2—C3^vi^	1.368 (7)
Ag2—O1^v^	2.615 (4)	C3—C5	1.474 (6)
Ag2—O4^i^	2.452 (3)	C2—H2	0.9300
O1—C4	1.298 (5)		
			
Ag1···C2	3.334 (5)	O1···O4^i^	3.154 (5)
Ag1···C4^i^	3.097 (4)	O2···C5	2.635 (6)
Ag1···Ag1^vii^	3.7449 (9)	O2···O3	3.072 (5)
Ag1···Ag1^viii^	3.7449 (9)	O2···O4	2.999 (5)
Ag1···Ag2^viii^	4.0452 (9)	O2···Ag2^xii^	3.666 (4)
Ag1···O1^viii^	4.022 (4)	O2···Ag2^i^	3.939 (4)
Ag1···O1^i^	3.495 (4)	O3···O2	3.072 (5)
Ag1···O3^i^	4.061 (3)	O3···Ag1^i^	4.061 (3)
Ag1···O3^iii^	3.320 (4)	O4···C4	3.268 (6)
Ag1···C5^i^	3.565 (4)	O4···Ag2^xiii^	4.026 (4)
Ag1···Ag2^ii^	3.6134 (9)	O4···O2	2.999 (5)
Ag1···C5^iii^	3.077 (5)	O4···Ag1^i^	3.031 (4)
Ag2···O1^i^	3.788 (4)	O4···O1^i^	3.155 (5)
Ag2···O4^ix^	4.026 (4)	O1···H2	2.5500
Ag2···C2^x^	3.898 (5)	O3···H2^xi^	2.5400
Ag2···Ag1^vii^	4.0452 (9)	C2···Ag1	3.334 (5)
Ag2···C2^i^	3.923 (5)	C2···Ag2^xiv^	3.898 (5)
Ag2···C1^i^	3.250 (5)	C2···Ag2^v^	3.928 (5)
Ag2···C2^v^	3.928 (5)	C2···Ag2^i^	3.923 (5)
Ag2···Ag1^xi^	3.6134 (9)	C4···O4	3.268 (6)
Ag2···C4^i^	3.458 (4)	C5···O2	2.635 (6)
Ag1···H2	3.0400	C5···Ag1^i^	3.565 (4)
Ag2···H2^v^	3.2300	H2···Ag1	3.0400
O1···O2	2.240 (5)	H2···O1	2.5500
O1···Ag2^i^	3.788 (4)	H2···Ag2^v^	3.2300
O1···Ag1^vii^	4.022 (4)	H2···O3^ii^	2.5400
O1···Ag1^i^	3.496 (4)		
			
Ag2^i^—Ag1—O1	88.36 (9)	Ag2—O1—Ag2^v^	88.62 (12)
O1—Ag1—O2^i^	103.99 (12)	Ag2^v^—O1—C4	114.3 (3)
O1—Ag1—O2^ii^	92.98 (12)	Ag1^i^—O2—C4	112.7 (3)
O1—Ag1—O4^iii^	151.46 (13)	Ag1^xi^—O2—C4	122.4 (3)
Ag2^i^—Ag1—O2^i^	68.57 (9)	Ag1^i^—O2—Ag1^xi^	104.81 (13)
Ag2^i^—Ag1—O2^ii^	162.28 (9)	Ag2^xii^—O3—C5	115.9 (3)
Ag2^i^—Ag1—O4^iii^	74.22 (9)	Ag2^i^—O4—C5	120.3 (3)
O2^i^—Ag1—O2^ii^	127.86 (12)	Ag1^xv^—O4—C5	119.1 (3)
O2^i^—Ag1—O4^iii^	90.69 (14)	Ag1^xv^—O4—Ag2^i^	119.34 (17)
O2^ii^—Ag1—O4^iii^	97.10 (13)	C2—C1—C3	120.9 (4)
O1—Ag2—O3^iv^	154.36 (13)	C2—C1—C4	117.4 (4)
O1—Ag2—O1^v^	91.37 (12)	C3—C1—C4	121.7 (4)
Ag1^i^—Ag2—O1	80.89 (9)	C1—C2—C3^vi^	117.2 (4)
O1—Ag2—O4^i^	82.89 (13)	C1—C3—C5	121.6 (4)
O1^v^—Ag2—O3^iv^	98.33 (13)	C1—C3—C2^vi^	121.8 (4)
Ag1^i^—Ag2—O3^iv^	78.00 (10)	C2^vi^—C3—C5	116.4 (4)
O3^iv^—Ag2—O4^i^	120.81 (13)	O1—C4—O2	125.3 (4)
Ag1^i^—Ag2—O1^v^	146.75 (8)	O1—C4—C1	120.8 (4)
O1^v^—Ag2—O4^i^	88.69 (12)	O2—C4—C1	114.0 (4)
Ag1^i^—Ag2—O4^i^	121.88 (9)	O3—C5—O4	127.9 (4)
Ag1—O1—Ag2	109.14 (14)	O3—C5—C3	118.1 (4)
Ag1—O1—C4	113.7 (3)	O4—C5—C3	114.0 (4)
Ag1—O1—Ag2^v^	116.43 (13)	C1—C2—H2	121.00
Ag2—O1—C4	111.9 (3)	C3^vi^—C2—H2	121.00
			
Ag2^i^—Ag1—O1—Ag2	−100.23 (13)	O1—Ag2—O1^v^—C4^v^	113.6 (3)
Ag2^i^—Ag1—O1—C4	25.5 (3)	O1—Ag2—O4^i^—C5^i^	125.7 (4)
Ag2^i^—Ag1—O1—Ag2^v^	161.49 (13)	Ag1—O1—C4—O2	−132.9 (4)
O2^i^—Ag1—O1—Ag2	−32.80 (17)	Ag1—O1—C4—C1	46.7 (5)
O2^i^—Ag1—O1—C4	92.9 (3)	Ag2—O1—C4—O2	−8.7 (6)
O2^i^—Ag1—O1—Ag2^v^	−131.09 (14)	Ag2—O1—C4—C1	170.9 (3)
O2^ii^—Ag1—O1—Ag2	97.46 (15)	Ag2^v^—O1—C4—O2	90.2 (5)
O2^ii^—Ag1—O1—C4	−136.8 (3)	Ag2^v^—O1—C4—C1	−90.3 (4)
O2^ii^—Ag1—O1—Ag2^v^	−0.82 (15)	Ag1^i^—O2—C4—O1	61.0 (5)
O4^iii^—Ag1—O1—Ag2	−151.8 (2)	Ag1^i^—O2—C4—C1	−118.5 (3)
O4^iii^—Ag1—O1—C4	−26.0 (5)	Ag1^xi^—O2—C4—O1	−65.4 (6)
O4^iii^—Ag1—O1—Ag2^v^	110.0 (3)	Ag1^xi^—O2—C4—C1	115.1 (4)
O1—Ag1—Ag2^i^—O4	−5.35 (14)	Ag2^xii^—O3—C5—O4	−31.4 (7)
O1—Ag1—Ag2^i^—O1^i^	70.34 (12)	Ag2^xii^—O3—C5—C3	151.0 (3)
O1—Ag1—Ag2^i^—O3^iii^	−124.33 (13)	Ag2^i^—O4—C5—O3	176.4 (4)
O2^i^—Ag1—Ag2^i^—O4	−111.08 (15)	Ag2^i^—O4—C5—C3	−5.9 (5)
O4^iii^—Ag1—Ag2^i^—O4	151.77 (16)	Ag1^xv^—O4—C5—O3	−16.2 (7)
O1—Ag1—O2^i^—C4^i^	−21.4 (3)	Ag1^xv^—O4—C5—C3	161.5 (3)
O1—Ag1—O2^ii^—C4^ii^	162.7 (3)	C3—C1—C2—C3^vi^	−0.1 (7)
O1—Ag1—O4^iii^—C5^iii^	17.6 (5)	C4—C1—C2—C3^vi^	179.2 (4)
O3^iv^—Ag2—O1—Ag1	129.8 (3)	C2—C1—C3—C5	−175.0 (4)
O3^iv^—Ag2—O1—C4	3.1 (5)	C2—C1—C3—C2^vi^	0.1 (7)
O3^iv^—Ag2—O1—Ag2^v^	−112.6 (3)	C4—C1—C3—C5	5.7 (7)
O1^v^—Ag2—O1—Ag1	−117.55 (14)	C4—C1—C3—C2^vi^	−179.2 (4)
O1^v^—Ag2—O1—C4	115.8 (3)	C2—C1—C4—O1	27.0 (6)
O1^v^—Ag2—O1—Ag2^v^	0.03 (10)	C2—C1—C4—O2	−153.5 (4)
Ag1^i^—Ag2—O1—Ag1	94.95 (13)	C3—C1—C4—O1	−153.8 (4)
Ag1^i^—Ag2—O1—C4	−31.8 (3)	C3—C1—C4—O2	25.8 (6)
Ag1^i^—Ag2—O1—Ag2^v^	−147.47 (8)	C1—C2—C3^vi^—C1^vi^	0.1 (7)
O4^i^—Ag2—O1—Ag1	−29.04 (15)	C1—C2—C3^vi^—C5^vi^	−175.3 (4)
O4^i^—Ag2—O1—C4	−155.7 (3)	C1—C3—C5—O3	−109.0 (5)
O4^i^—Ag2—O1—Ag2^v^	88.54 (12)	C1—C3—C5—O4	73.0 (6)
O1—Ag2—O3^iv^—C5^iv^	8.6 (6)	C2^vi^—C3—C5—O3	75.6 (6)
O1—Ag2—O1^v^—Ag1^v^	−110.74 (15)	C2^vi^—C3—C5—O4	−102.3 (5)
O1—Ag2—O1^v^—Ag2^v^	−0.03 (11)		

Symmetry codes: (i) −*x*, −*y*+1, −*z*+1; (ii) *x*, −*y*+1/2, *z*+1/2; (iii) *x*, −*y*+3/2, *z*+1/2; (iv) −*x*, *y*−1/2, −*z*+1/2; (v) −*x*, −*y*, −*z*+1; (vi) −*x*+1, −*y*+1, −*z*+1; (vii) −*x*, *y*−1/2, −*z*+3/2; (viii) −*x*, *y*+1/2, −*z*+3/2; (ix) *x*, *y*−1, *z*; (x) *x*−1, *y*, *z*; (xi) *x*, −*y*+1/2, *z*−1/2; (xii) −*x*, *y*+1/2, −*z*+1/2; (xiii) *x*, *y*+1, *z*; (xiv) *x*+1, *y*, *z*; (xv) *x*, −*y*+3/2, *z*−1/2.

### Hydrogen-bond geometry (Å, °)

**Table:**

*D*—H···*A*	*D*—H	H···*A*	*D*···*A*	*D*—H···*A*
C2—H2···O3^ii^	0.9300	2.5400	3.422 (6)	158.00

Symmetry codes: (ii) *x*, −*y*+1/2, *z*+1/2.
